# Analysis of TERT mRNA Levels and Clinicopathological Features in Patients with Peritoneal Mesothelioma

**DOI:** 10.3390/cancers17020252

**Published:** 2025-01-14

**Authors:** Antonio d’Amati, Gabriella Serio, Andrea Quaranta, Luigi Vimercati, Michelina De Giorgis, Loredana Lorusso, Mariella Errede, Vito Longo, Andrea Marzullo, Domenico Ribatti, Tiziana Annese

**Affiliations:** 1 Department of Medicine and Surgery, LUM University, Casamassima, 70010 Bari, Italy; damati@lum.it; 2 Department of Translational Biomedicine and Neuroscience, University of Bari Medical School, 70124 Bari, Italy; michelina.degiorgis@uniba.it (M.D.G.); loredana.lorusso@uniba.it (L.L.); mariella.errede@uniba.it (M.E.); domenico.ribatti@uniba.it (D.R.); 3 Department of Precision and Regenerative Medicine and Ionian Area, Pathology Unit, University of Bari “Aldo Moro”, 70124 Bari, Italy; gabriella.serio1@uniba.it (G.S.); a.quaranta35@studenti.uniba.it (A.Q.); andrea.marzullo@uniba.it (A.M.); 4 Department of Interdisciplinary Medicine, Occupational Health Unit, University of Bari “Aldo Moro”, 70124 Bari, Italy; luigi.vimercati@uniba.it; 5 Thoracic Oncology Unit, IRCCS Istituto Tumori “Giovanni Paolo II”, 70124 Bari, Italy; v.longo@oncologico.bari.it

**Keywords:** BAP1, inflammatory infiltrate, malignant peritoneal mesothelioma, MTAP, p16, RNAscope, TERT mRNA

## Abstract

Background/Objectives: Telomerase reverse transcriptase (TERT) is the catalytic subunit of the telomerase enzyme responsible for telomere length maintenance and is an important cancer hallmark. Our study aimed to clarify the mRNA expression of TERT in peritoneal mesothelioma (PeM), and to explore the relationship between its expression and the clinicopathological parameters and prognosis of patients with PeM. Methods: In a cohort of 13 MpeM patients, we evaluated histotype, nuclear grade, mitotic count, necrosis, inflammation, Ki67, BAP1, MTAP and p16 expression by immunohistochemistry, *p16*/*CDKN2A* status by FISH and TERT mRNA expression by RNAscope. Results: Our results showed several statistical correlations between TERT mRNA-score and other investigated features: (i) a poor positive correlation with BAP1 score (*r* = 0.06340; *p* ≤ 0.0001); (ii) a moderate positive correlation with *p16* FISH del homo (*r* = 0.6340; *p* ≤ 0.0001); (iii) a fair negative correlation with *p16* FISH del hetero (*r* = −0.3965; *p* ≤ 0.0001); a negative poor correlation with MTAP (*r* = −0.2443; *p* ≤ 0.0001); and (iv) a negative fair correlation with inflammatory infiltrate (r = −0.5407; *p* = 0.0233). Moreover, patients survive for a significantly longer time if they have a low mitotic index adjusted (2–4 mitotic figures per 2 mm^2^) (*p* ≤ 0.0001), are male (*p* = 0.0152), lose BAP1 (*p* = 0.0152), are p16 positive and present no deletion or heterozygous for *p16* (*p* ≤ 0.01). Conclusions: TERT is highly expressed in PeM, but it is not one of the crucial factors in evaluating the prognosis of patients. Nevertheless, the results validate the prognostic significance of the mitotic index, BAP1 loss and *p16*/CDKN2A status.

## Simple Summary

Peritoneal mesothelioma (PeM) is a rare, aggressive cancer with globally rising mortality rates. Its diagnosis is challenging due to a lack of specific clinical signs, relying primarily on peritoneal biopsy and immunohistochemical staining. Patients face a 5-year survival rate of around 5%, highlighting the urgent need for early detection and treatment. Our study provides a detailed morphological and molecular perspective on the PeM heterogeneity. Results validate the prognostic significance of the mitotic index, BAP1 loss and *p16*/CDKN2A status, highlighting meaningful differences in survival outcomes and emphasizing the relevance of morphological and molecular alterations in clinical practice. Moreover, several statistical correlations between the TERT mRNA level and other investigated morphological and molecular features were observed.

## 1. Introduction

Peritoneal mesothelioma (PeM) is an aggressive and uncommon cancer of the abdominal cavity, comprising up to 30% of all mesotheliomas [1]. PeM is divided into three histopathological subtypes: epithelioid (80% of PeM), sarcomatoid (rare) and biphasic/mixed (13% of PeM) [2]. Epithelioid is the least aggressive among the three types and is associated with the most favorable outcomes. Sarcomatoid and biphasic tumors are more infiltrative and related to poorer outcomes [3]. Sarcomatoid is defined by a dense growth of spindle cells arranged in fascicles and shows a higher degree of cellular abnormality [4]. The biphasic subtype comprises at least 10% of epithelioid and sarcomatoid cellular features. In the past, patients were treated with systemic therapy with limited success [5]. More recently, cytoreductive surgery [6] and hyperthermic intraperitoneal chemotherapy [7] have been employed with substantial enhancements in survival rates [8,9]. In the frontline for disease management, immunotherapy, either alone or in conjunction with chemotherapy, has gained much attention in treating inoperable PeM [10]. The diagnosis of PeM is obtained after a comprehensive evaluation that includes medical history, physical examination, laboratory tests, radiographic investigations, and diagnostic laparoscopy with histopathological diagnosis on several peritoneal biopsies [11,12].

Concerning PeM pathogenesis, the connection with asbestos exposure is not as strong as with pleural mesothelioma, but the suggested rationale for the development of the disease is the same. Exposure to the carcinogen is believed to lead to the accumulation of tiny fiber particles that cause harm. One of the proposed pathogenetic mechanisms is the emission of ROS and RNS (reactive oxygen and nitrogen species) by mesothelial and inflammatory cells that damage DNA, leading to genetic instability and triggering an inflammatory response by attracting various chemicals and proteins to promote cell growth and cancer development [13,14]. Recently, in patients without asbestos exposure, loss of chromosome regions and tumor suppressor genes (TSGs), as well as gain of function mutations in oncogenes, were also linked to the onset of PeM [15,16,17]. The most frequently altered genes in PeM were *BAP1*, *NF2*, *CDKN2A*, *CDKN2B* and *PBRM1* [16,18,19].

Telomerase reverse transcriptase (TERT) is the catalytic subunit of the enzyme telomerase responsible for telomere length maintenance [20,21]. TERT is an important cancer hallmark [22], primarily known for maintaining telomere length by preventing cellular senescence and ensuring immortality [23], but it also has telomere-independent roles responsible for tumor progression. It rewires tumor metabolism [24,25,26,27], as in glioblastoma [24,28], and promotes tumor initiation or progression, or both [29,30,31]. In a panel of alternative lengthening of telomeres (ALT) cells with recombinant expression of TERT and its variants, it was demonstrated that TERT expression provides protection against double-strand DNA damage separately from its involvement in telomere synthesis, and that it enhances the proliferation of oncogene-transformed cells by mitigating the inhibitory effects of telomere attrition and genotoxic stress generated by chemicals or metabolism [32]. TERT also affects gene expression by interacting with chromatin remodeling factors, regulating DNA methylation and activating its RNA-dependent RNA Polymerase (RdRP) to produce siRNAs [33]. It also exerts its function on the ubiquitin-proteasomal system, upregulating 26S proteasome activity and assembly, providing cytoprotective effects against apoptosis induced by proteasome dysfunction [34,35]. Moreover, it is involved in tumor invasiveness, promoting the local invasion of primary tumor cells, intravasation, survival of circulating tumor cells, extravasation, micrometastasis formation and macrometastatic growth [36]. Among the non-canonical biological functions of TERT, it influences different components of the tumor microenvironment, including inflammatory factors and the immune response [37,38]. Several studies have demonstrated that TERT mRNA levels in tumors are potential diagnostic and prognostic markers [39,40,41,42]. In colorectal cancer [43,44] and metastatic melanoma [45], tissue and plasma TERT mRNA levels were found to be independent markers of therapeutical response and, therefore, were prognostic of disease progression.

The present study aimed to evaluate TERT mRNA expression in PeM by RNAscope and correlate its levels with morphological and molecular factors that are currently used for prognosis and patient survival. We evaluated histotype, nuclear grade, mitotic count, necrosis, inflammation, Ki67, BAP1 and MTAP protein expression by immunohistochemistry, p16 protein expression by immunohistochemistry and its deletion by FISH.

## 2. Materials and Methods

### 2.1. Patients and Tissue Collection

This study was performed following the Declaration of Helsinki on archival material belonging to the project, which was reviewed and approved by the Ethics Committee of the University of Bari Aldo Moro (Accession number 5062, 22 June 2016).

Our study population consisted of archival tissue samples from 13 patients with peritoneal mesothelioma collected in 2003–2017. Information on patients’ sex, age, survival time (as months between the histological diagnosis and the date of death), asbestos exposure, and year of exposure were gathered via questionnaires and categorized based on the National Mesothelioma Register criteria (COR Apulia) (Table 1).

The biopsy specimens—formalin-fixed and paraffin-embedded—were cut into a series of sections (3 to 5 microns) used for all the morphological and molecular analyses. Histological sections were stained with hematoxylin-eosin and evaluated for the following: histotype (epithelioid, biphasic and sarcomatoid); nuclear atypia (from 1 (mild) to 3 (severe)); inflammatory infiltrate (scored as 1, low; 2, moderate; or 3, extensive) (Table 2); the presence of necrosis; mitotic count (score of 1, 2 or 3 based on mitoses in 1, 2–4 and ≥5, expressed on 2 mm^2^ and indicated as per the guidelines for grading in epithelioid pleural mesothelioma) [46]; or score of 1, 2 or 3 based on mitoses in 2–4, 5–9 and >10 expressed on 2 mm^2^ [47]); nuclear grade (score of 1–3 was assigned: 1, round uniform nuclei; 2, irregular nuclear contours, small nucleoli; and 3, pleomorphism and markedly irregular nuclear contours and prominent nucleoli [47]); immunostaining for Ki-67, BAP1, p16 and MTAP; *p16* deletion by FISH; TERT mRNA expression by RNAscope.

### 2.2. Tert RNAscope

Sections of 4-μm from all FFPE tissue samples were cut on a microtome and mounted on Superfrost Plus slides (Thermo Fisher Scientific, Waltham, MA, USA). The slides were baked in the HybEZTM Oven (Advanced Cell Diagnostics (ACD), Hayward, CA, USA) for 60 min at 60 °C, deparaffinized via incubating in xylene followed by 100% ethanol, and then pretreated according to the manufacturer’s instructions for manual assay using the RNAscope^®^ 2.5 HD Reagent Kit (ref. 322360, Advanced Cell Diagnostics (ACD), Hayward, CA, USA). In brief, the pretreatment consisted of submerging the slides into pretreat-1 solution for 10 min, pretreat-2 for 15 min, and pretreat-3 for 30 min (ref. Pretreatment kit 322330, Advanced Cell Diagnostics (ACD), Hayward, CA, USA). The slides were then covered with the RNAscope^®^ Probe–Hs-TERT-O1–Homo sapiens telomerase reverse transcriptase (TERT) transcript variant 1 mRNA (ref. 481961, Advanced Cell Diagnostics (ACD), Hayward, CA, USA), Hs-PPIB positive control probe (ref. 313901, Advanced Cell Diagnostics (ACD), Hayward, CA, USA) or negative control DapB probe (ref. 310043, Advanced Cell Diagnostics (ACD), Hayward, CA, USA) at 40 °C for 2 h. The Hs-PPIB probe was used as a control to ensure RNA quality. After the probes’ hybridizations, sections were subjected to signal amplification and detection using a Fast-RED solution.

Images of the whole slides were captured using the scanning platform Aperio ScanScope CS (Leica Biosystems, Nussloch, Germany) at a magnification of ×40. TERT mRNA semiquantitative analysis was performed on 6 randomly selected 0.12 mm^2^ layers per slide by an expert pathologist using the Aperio RNA ISH algorithm embedded in the ImageScope v.11.2.0.780 (Leica Biosystems, Nussloch, Germany). The obtained results were divided into five customized ranges/scores: 0, which includes cells that do not contain dots per cell; 1+, which includes cells containing 1–3 dots per cell; 2+, which includes cells containing 4–9 dots per cell; 3+, which includes cells containing 10–15 dots per cell; and 4+ which includes cells containing ≥16 dots per cell. Results were reported as mean ± sd. Statistical analysis was performed using two-way ANOVA analysis and Tukey’s multiple comparisons test with GraphPad Prism 7.04. *p* ≤ 0.05 was considered the limit for statistical significance.

### 2.3. BAP1, P16, MTAP, Ki-67 Immunohistochemistry

A broad panel of antibodies for immunohistochemistry was used, following WHO recommendations [48].

BRCA1-associated protein 1 (BAP1) (clone C4, Santa Cruz Biotechnology, Santa Cruz, CA, USA) was classified as negative only if a complete absence of nuclear staining was observed in the presence of nuclear-positive lymphocytes.

P16 was analyzed using a monoclonal antibody (clone G175-405, BD Pharmingen; 1:50 dilution) and was assessed for the number of positive cells and the strength of cytoplasmic and/or nuclear staining (score 0: absent, score 1: focal or diffuse low-intensity staining, score 2: diffuse and moderate intensity staining, score 3: diffuse and high-intensity staining). P16 was deemed positive only for scores ≥ 2 [49].

MTAP protein expression was analyzed using a monoclonal antibody (clone 2G4 from Abnova Corp., Taipei City, Taiwan; 1:200 dilution). In accordance with previous similar studies, such as by Chapel et al. [50] and Hida et al. [51], a value of 1 was assigned to tumors that showed a complete loss of MTAP expression or a “faint” cytoplasmic positivity, and a value of 2 to tumors that showed “strong” cytoplasmic positivity in more than 1% of the neoplastic cells. Immunoreactivity was considered “strong” when tumor cells stained more intensely than the internal control (e.g., lymphocytes, endothelial cells, fibroblasts) and “faint” when they stained less intensely than the internal control. Cases without sufficient tumor tissue for evaluation or with a failed positive internal control were excluded from further analysis.

Ki67/MIB1 was evaluated (clone K5001, DAKO, Denemark, dilution 1:200) and was expressed as the percentage of positive cells in a total of at least 1000 cells. Positivity was also classified as either low (≤20%) or high (>20%) based on the median value.

### 2.4. Fluorescence In Situ Hybridization (FISH)

*P16*/cyclin-dependent kinase inhibitor 2A (*CDKN2A*) deletion was investigated using a dual color FISH locus-specific *CDKN2A* (9p21) commercially available probe (Abbott, Abbott Park, IL, USA) to detect chromosome 9 deletion [52]. A FISH locus-specific *CDKN2A* (9p21) probe (Abbott, Abbott Park, IL, USA) was used to detect chromosome 9 deletion. Vysis LSI *CDKN2A*/CEP 9 probes are provided in one vial as a mixture of the LSI *CDKN2A* (*p16*) probe, labeled with Spectrum Orange, and the CEP 9 probe, labeled with Spectrum Green. The LSI *CDKN2A* probe spans approximately 222 kb and contains several genetic loci, including *D9S1749*, *DS1747*, *p16* (*INK4B*), *p14* (*ARF*), *D9S1748*, *p15* (*INK4B*) and *D9S1752*. The CEP 9 Spectrum Green probe hybridizes to alpha satellite sequences specific to chromosome 9, CE Marked. A minimum of 100 non-overlapped intact interphase nuclei of consecutive cells were scored for each case. A cut-off of 20% was used for homozygous deletion.

The heterozygous deletion was defined as occurring when >20% of the cells showed only one signal or a lower signal number than CEP9.

### 2.5. Statistical Analyses

Statistical analyses were performed in GraphPad Prism version 7.04 for Windows (GraphPad Software, La Jolla, CA, USA). The distribution of datasets was assessed using D’Agostino and Pearson Omnibus and Shapiro–Wilk normality tests.

Two-way ANOVA with post-hoc Tukey’s multiple comparisons test was used to calculate the significance level of the difference in TERT mRNA expression between the samples. Spearman nonparametric correlation analysis was used to assess the association between TERT mRNA expression and the other morphological and cytogenetic parameters. Survival curves were calculated using the Kaplan–Meier method and significance was assessed using the log-rank (Mantel–Cox) test. *p* values less than 0.05 in the two-tailed analyses were considered significant and are summarized in Figure panels as * *p* ≤ 0.05; ** *p* ≤ 0.01; *** *p* ≤ 0.001; **** *p* ≤ 0.0001.

## 3. Results

Thirteen patients, 11 male (84.6%) and 2 female (15.4%), with a mean age of 66.5 ± 8.6 years, were included in the study. Asbestos exposure (occupational and/or environmental) was known in 9 of the 13 patients (69.2%). Clinical data were collected for all patients (Table 1).

### 3.1. Histologic Evaluation

**Figure 1:**
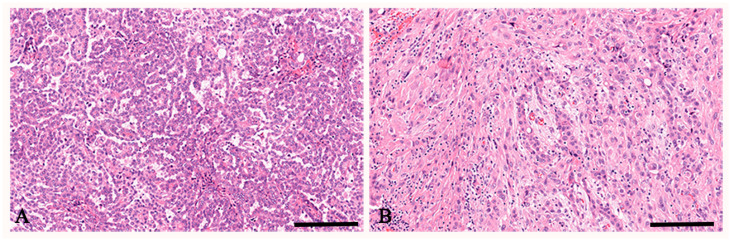
Hematoxylin and eosin staining of peritoneal mesothelioma. In (**A**), a representative case of peritoneal mesothelioma, epithelioid histotype is observed. In (**B**), a representative case of peritoneal mesothelioma, biphasic histotype is observed. Scale bar: (**A**,**B**) 112 μm.

Tumor histotypes included seven (53.9%) epithelioid and six (46.1%) biphasic mesotheliomas (Table 1). Representative photomicrographs of epithelioid and biphasic mesotheliomas are shown in Figure 1.

Nuclear grade 1 was reported in one case (7.7%), grade 2 in five cases (38.5%), and grade 3 in seven cases (53.8%) (Table 3). Mitotic count was >10/2 mm^2^ in one case (14.3%) of epithelioid and four cases (66.6%) of biphasic mesothelioma, respectively (Table 3). Necrosis was observed in seven cases (53.8%): two (28.57%) epithelioid and five (71.43%) biphasic mesotheliomas. Inflammatory infiltrate was present in eight cases (61.5%): five (71.43%) epithelioid and three (50%) biphasic mesotheliomas. Desmoplastic stromal reaction was observable in six cases (46.2%): one (17.77%) epithelioid and five (83.33%) biphasic mesotheliomas, respectively. Ki67 value was >20% in two cases (28.6%) of epithelioid and four cases (66.6%) of biphasic histotypes (Table 3).

### 3.2. BAP1 Expression

In accordance with previous observations in a large cohort of mesotheliomas [53] (frequent loss of BAP1 expression, particularly in the epithelioid subtype), our immunohistochemical evaluation for BAP1 showed negativity in all seven (100%) cases of epithelioid and in two cases (33.3%) of biphasic mesotheliomas (Table 4).

### 3.3. P16 Expression and CDKN2A Status

Immunohistochemical evaluation for the p16 protein showed positivity in five cases (71.4%) and two cases (33.3%) of epithelioid and biphasic mesotheliomas, respectively (Table 4). FISH analysis for *p16*/*CDKN2A* revealed the absence of deletion in one case (14.3%) of epithelioid and one case (16.7%) of biphasic mesotheliomas, heterozygous deletion in three cases (42.8%) of epithelioid and one case (16.7%) of biphasic mesotheliomas, and homozygous deletion in three cases (42.8%) and four cases (66.7%) of epithelioid and biphasic mesotheliomas, respectively.

### 3.4. MTAP Expression

Immunohistochemical evaluation for the MTAP protein showed complete negativity in two cases of epithelioid (28.6%) and two cases of biphasic (33.3%) mesotheliomas (Table 4). Two cases (28.6%) of epithelioid and three cases (50%) of biphasic mesotheliomas, respectively, showed “faint” positivity (i.e., lower than the internal control) and were considered negative (i.e., MTAP loss of expression). On the other hand, three cases (42.8%) and one case (16.7%) of epithelioid and biphasic mesotheliomas, respectively, showed “strong” cytoplasmic positivity; hence, MTAP expression was considered retained.

### 3.5. TERT

**Figure 2:**
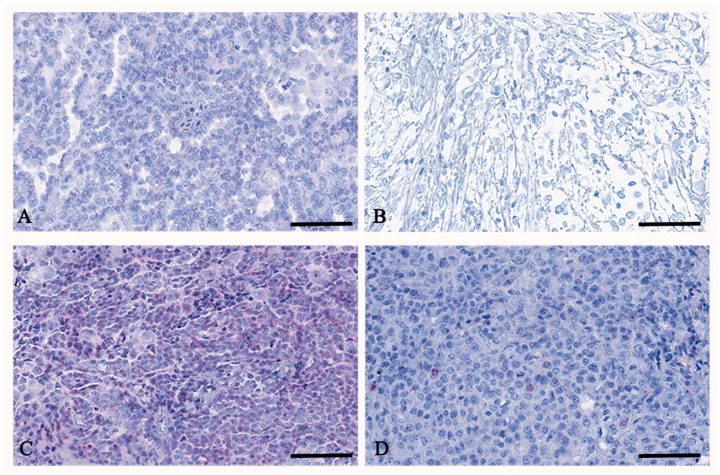
RNAscope of TERT on peritoneal mesothelioma. In (**A**), a representative case of peritoneal mesothelioma, epithelioid histotype, shows moderate TERT expression of up to 9 dots per cell. In (**B**), a representative case of peritoneal mesothelioma, biphasic histotype, shows low TERT expression of up to 3 dots per cell. In (**C**), a representative case of peritoneal mesothelioma, epithelioid histotype, shows high TERT expression of more than 16 dots per cell. In (**D**), a representative case of peritoneal mesothelioma, biphasic histotype, shows high TERT expression of up to 9 dots per cell. In all cases, TERT is found as usual in the nuclei but also in the cytoplasm. Scale bar: (**A**–**D**) 56 μm.

**Figure 3:**
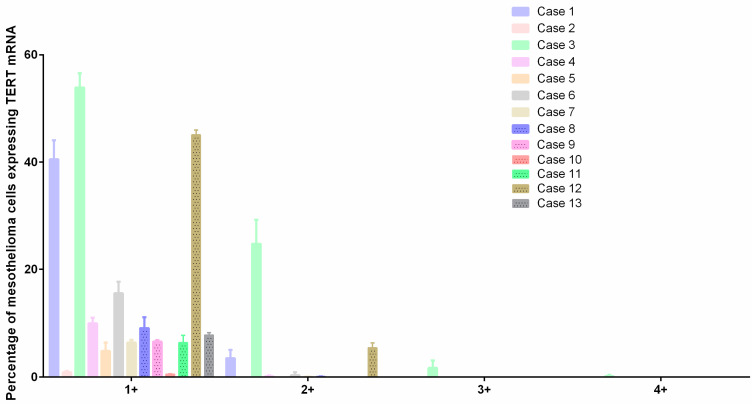
TERT mRNA expression by RNAscope. Interleaved bar shows TERT mRNA expression in all the samples divided into five customized ranges: 0, which includes cells that do not contain dots per cell (not represented); 1+, which includes cells containing 1–3 dots per cell; 2+, which includes cells containing 4–9 dots per cell; 3+, which includes cells containing 10–15 dots per cell; and 4+, which includes cells containing ≥16 dots per cell.

The TERT mRNA expression quantification, represented as red dots (Figure 2), in malignant peritoneal mesothelioma showed a different expression panel among the different histotypes (Figure 3, Table 5).

In general, all cases presented an overexpression of TERT, but for some, there was an overexpression with cells showing more than 16 molecules/dots per cell. There was a mean of 23.9 ± 9 and 6.6 ± 6.4 percent of cells containing 1–3 dots per cell in epithelioid and biphasic mesotheliomas, respectively. Four cases (57.1%) of epithelioid and three cases (50%) of biphasic mesotheliomas showed cells containing 4–9 dots per cell, and only one case (14.3%) of epithelioid mesothelioma showed cells with 10–15 dots per cell and cells with ≥16 dots per cell.

**Figure 4:**
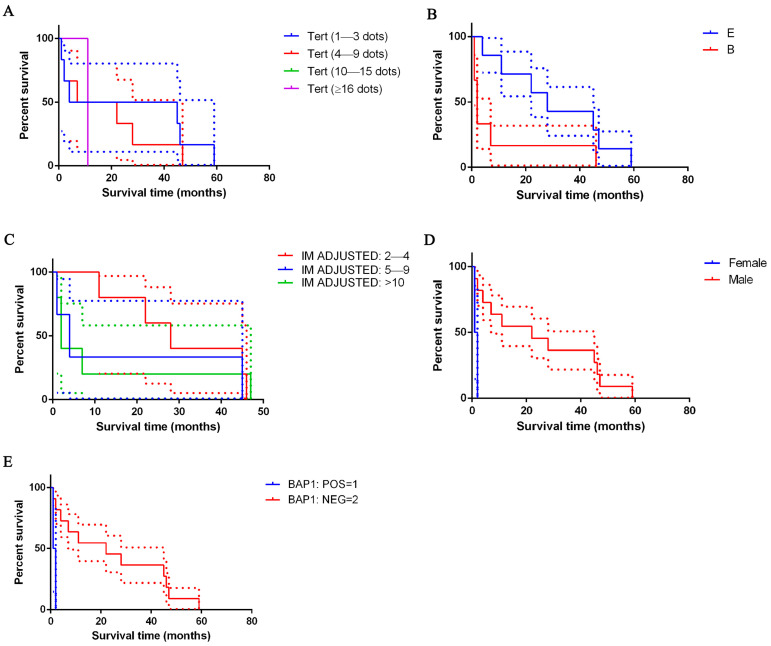
Kaplan–Meier survival of patients with peritoneal mesothelioma. In (**A**), survival data is shown based on TERT score: patients with high TERT mRNA expression showed shorter survival times than their counterparts (violet lines); not statistically significant. In (**B**), survival data is shown based on histological type: epithelioid type (line blue), biphasic type (line red); differences are not significant. In (**C**), survival data based on mitotic index adjusted is shown: 2–4 (line red), 5–9 (line blue) or >10 (line green) mitosis expressed on 2 mm^2^; *p* ≤ 0.0001. In (**D**), survival data based on sex is shown: male (line blue) and female (line red); *p* = 0.0152. In (**E**), survival data based on BAP1 is shown: positive (line blue) and negative (line orange); *p* = 0.0152. The dashed lines indicate SE.

No statistically significant difference was detected as regards survival in correlation with TERT mRNA expression (Figure 4), although the slope for the range 4+, which includes cells containing ≥16 dots per cell, was steeper, indicating a higher event rate (death rate) and, therefore, a possible worse survival prognosis. On the contrary, a flatter slope for 1+, which includes cells containing 1–3 dots per cell, was highlighted, indicating a lower event rate and, therefore, a better survival prognosis.

**Figure 5:**
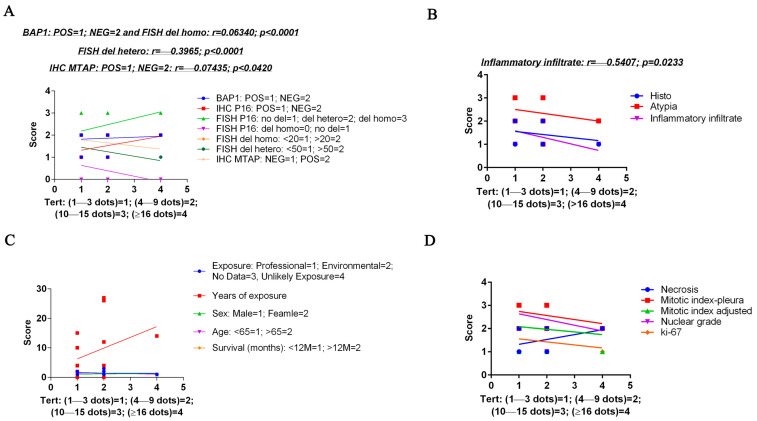
Spearman’s correlation graphs between the customized score relative to the percentage of cells containing TERT mRNA (1+–4+ on the *x*-axis) and the score for a series of parameters obtained from morphological and cytogenetic analyses (*y*-axis). In (**A**), TERT mRNA correlation with BAP1, p16 and MTAP protein expression, and with *p16* cytogenetic alterations. In (**B**), TERT mRNA correlation with histotype, atypia and inflammatory infiltrate. In (**C**), TERT mRNA correlation with asbestous exposure type and time, sex, age and survival month. In (**D**), TERT mRNA correlation with necrosis, mitotic indexes, nuclear grade and ki-67. Statistical significance was determined by linear regression analysis. Only the significant r and *p*-values are reported.

Our results (Figure 5) showed several statistical correlations among TERT mRNA-scores and other investigated features: (i) a poor positive correlation with BAP1 score (r = 0.06340; *p* ≤ 0.0001); (ii) a moderate positive correlation with *p16* FISH del homo score (r = 0.6340; *p* ≤ 0.0001); (iii) a fair negative correlation with *p16* FISH del hetero (r = −0.3965; *p* ≤ 0.0001); a negative poor correlation with MTAP score (r = −0.07435; *p* = 0.0420); and (iv) negative fair correlation with inflammatory infiltrate (r = −0.5407; *p* = 0.0233).

No correlations were found with the type of exposure, the years of exposure, the sex, the age score and survival, or with necrosis, mitotic index, nuclear grade and ki-67 (Figure 5).

### 3.6. Analysis of Survival

Taking all the cases of PeM together, patients’ survival ranged between 1 and 59 months (Table 1). For the epithelioid type, mean survival was 30.9 ± 20.2 months (range 4–59 months; CI: 12.2–49.6; median survival time 28 months); for the biphasic type, it was 9.8 ± 17.9 months (range 1–46; CI: −8.9–28.58; median survival time 2 months). Patients with epithelioid mesothelioma survived longer than those with a biphasic histotype (Figure 4).

Cases with lower adjusted mitotic index (mitoses in 2–4 cells) (*p* ≤ 0.0001), males (*p* = 0.0152), those with the loss of BAP1 (*p* = 0.0152), positivity for p16 in immunohistochemistry, *p16* heterozygous deletion or no *p16* deletion (depending on the employed score) (*p* ≤ 0.0001) showed better survival than their counterparts (Figure 4; Table 6).

## 4. Discussion

In the present single-center retrospective study on 13 patients affected by PeM, several morphological and molecular parameters were analyzed and correlated with TERT mRNA expression and survival in order to better understand the role of TERT and its relationships with the clinicopathological features of our cohort.

In tumors, the common result of the diverse mechanisms leading to the activation of TERT is an increase in TERT mRNA expression. The quantification of TERT mRNA may serve as a prognostic indicator in many different tumors, such as thyroid carcinoma [54,55], Wilm’s tumor [56], non-small cell lung cancers [57] and neuroblastoma [58].

Here, TERT mRNA expression was evaluated by RNAscope. Among the quantitative (real-time PCR) and qualitative (in situ hybridization) techniques for transcript analyses, RNAscope is a valid high-throughput method [59]. RNAscope assay enables the association between histomorphology and gene expression, accurately distinguishing between neoplastic and non-neoplastic tissues, making it a helpful diagnostic tool [60,61,62].

The use of the RNAscope assay to assess TERT status in FFPE tissue has already been evaluated and validated in tumor samples [63]. However, in our study, we used a specific alternative algorithm to quantify the mRNA signals (Aperio RNA ISH algorithm embedded in the ImageScope), and no data on PeM have previously been reported in the literature.

Although not significant, we found an increased TERT mRNA expression in epithelioid PeM cases compared to biphasic ones. Since our results reported that in the epithelioid histotype, the mitotic index is generally lower than in the biphasic one, while the presence of inflammatory infiltrate is more evident, the expression of TERT in epithelioid PeM could be related to the activation of non-canonical mechanisms, such as the recruitment and maintenance of inflammation. It has been reported that TERT has effects on inflammation and the immune response. For instance, the production of inflammatory cytokines IL-6 and TNF-α, which play a crucial role in cancer-related inflammation, depends also on TERT binding to NF-κB [64,65]. The expression of inflammatory markers INFγ, TNF, IL10 and PD-1 in a non-small-cell lung cancer mouse model was influenced by TERT [66]. Moreover, TERT is recognized by T cells and can induce an adaptive immune response [67]. For instance, TERT is required for melanoma progression, avoiding immune rejection and regression. [68]. In CD4+ T cell activation, TERT is transported from the cytoplasm to the nucleus via kinase Akt [69].

In contrast to a study by Xian et al. [70], we found a negative fair correlation between TERT expression and inflammatory infiltrate. A plausible explanation lies in the diversity between mesothelioma and head and neck tumors and their microenvironments. We believe that further studies will clarify the role of TERT in the PeM inflammatory microenvironment.

Moreover, we have evaluated the correlation between TERT expression and several features: histotype, nuclear grade, mitotic count, necrosis, inflammation, Ki67, BAP1 and MTAP protein expression by immunohistochemistry, p16 protein expression by immunohistochemistry and its deletion (CDKN2A gene) by FISH. Our results showed several statistical correlations among TERT mRNA-scores and other investigated features: (i) a poor positive correlation with BAP1 score (r = 0.06340; *p* ≤ 0.0001); (ii) a moderate positive correlation with *p16* FISH del homo (r = 0.6340; *p* ≤ 0.0001); (iii) a fair negative correlation with *p16* FISH del hetero (r = −0.3965; *p* ≤ 0.0001); a negative poor correlation with MTAP score (r = −0.2443; *p* ≤ 0.0001); and (iv) a negative fair correlation with inflammatory infiltrate (r = −0.5407; *p* = 0.0233).

A lower mitotic index (2–4 mitotic figures per 2 mm^2^) (*p* ≤ 0.0001), male sex (*p* = 0.0152), BAP1 loss (*p* = 0.0152), positivity for p16 by immunohistochemistry, CDKN2A heterozygous deletion or no CDKN2A deletion (depending on the employed score) (*p* ≤ 0.0001) predict for better overall survival independently of other parameters.

Previous studies have examined the prognostic significance of the mitotic index in peritoneal mesothelioma; univariate analysis showed that more prolonged overall survival was significantly associated with a low mitotic index [71,72].

In the United States and Europe, PeM incidence is higher in males vs. females [73,74]; in our cohort, 85% of enrolled patients were males. However, our data demonstrate an inverse trend because we found better overall survival in males vs. females [71,75], probably due to our small cohort size and because men often entered follow-up earlier than females due to anamnestic data of occupational asbestos exposure.

In accordance with the existing literature, we found a loss of BAP1 nuclear protein expression in 85% of our cases [18], and our data confirmed a better overall survival rate for patients with BAP1 protein loss independently of other clinicopathological features [72,76,77,78,79]. BAP1, as a tumor suppressor, is involved in chromatin remodeling and gene expression regulation. Emerging evidence (particularly for hepatocellular carcinoma) suggests that TERT could interact with BAP1 via the WNT/β-catenin signaling pathway, which is often activated in tumors with TERT overexpression. This interaction might disrupt BAP1’s ability to maintain genomic stability, facilitating tumor progression. Furthermore, TERT’s telomerase-independent functions, such as its role in modulating transcriptional networks, could influence BAP1 activity by altering the epigenetic landscape [80,81].

The results of immunohistochemistry for p16 expression and FISH analysis *p16*/CDKN2A status showed that 54% of our cases were positive for p16 protein expression and that 15% did not present CDKN2A deletions, while its homozygous deletion was found in 54% of cases and heterozygous deletion in 31%. As previously demonstrated by our group, confirmed by the results of this study and consistent with the literature, no deletion/heterozygous deletion of *p16*/CDKN2A resulted in the most prolonged survival [72,78]. Moreover, our data showed better overall survival in patients with p16 positivity by immunohistochemistry, in accordance with Borczuk [82], which demonstrated that p16 loss was independently linked to poorer survival outcomes. TERT expression has been linked to cell cycle regulation, where it may downregulate tumor suppressors like p16 through the activation of the PI3K/AKT/mTOR pathway. This pathway is known to promote cellular proliferation and bypass senescence mechanisms driven by p16 loss. Additionally, TERT’s role in telomere maintenance can indirectly influence p16 levels by mitigating telomere dysfunction, which otherwise triggers a p16-mediated senescence response [83].

## 5. Conclusions

This study enhances the understanding of PeM by exploring its morphological and molecular heterogeneity, emphasizing the role of specific markers—the mitotic index, BAP1 loss, and *p16*/CDKN2A status—in prognostic accuracy and clinical decision-making. The interplay between TERT, p16 and BAP1 likely creates a permissive environment for tumor development by promoting immortalization (via telomere elongation), evasion of growth suppression (via p16 inhibition) and genomic instability (via BAP1 inactivation). Although these mechanisms are supported by the current literature, further experimental studies, such as pathway-specific inhibition or knockdown models, would be required to definitively elucidate the molecular crosstalk. Despite achieving statistical significance, several limitations must be acknowledged: the cohort size was limited, the study lacked samples from the sarcomatoid subgroup and the retrospective nature of our analysis restricts the strength of our conclusions. Larger, prospective studies are needed to confirm these results and evaluate additional markers like TERT, which may improve clinical outcome stratification beyond histological features alone.

## Figures and Tables

**Table 1:** Demographic characteristics of mesothelioma patients.

Patients	Asbestos Exposure	Year of Exposure	Sex	Age	Survival Time (Months)
Case 1	1	4	M	64	47
Case 2	1	4	M	58	2
Case 3	1	14	M	59	11
Case 4	1	26	M	64	7
Case 5	2	Not countable	M	68	4
Case 6	2	12	F	72	1
Case 7	3	-	F	80	2
Case 8	2	Not countable	M	50	28
Case 9	2	15	M	72	46
Case 10	1	10	M	59	1
Case 11	1	Not countable	M	72	59
Case 12	1	27	M	68	22
Case 13	1	2	M	78	45

For asbestos exposure score means: 1 = professional; 2 = environmental; 3 = no data.

**Table 2:** Morphological characteristics of mesothelioma patients’ biopsies relative to cell type.

Patients	Histotype	Atypie	Inflammatory Infiltrate
Case 1	1	1	1
Case 2	2	3	2
Case 3	1	2	1
Case 4	2	3	1
Case 5	1	2	2
Case 6	2	3	1
Case 7	2	3	2
Case 8	1	2	1
Case 9	2	3	1
Case 10	2	3	2
Case 11	1	2	1
Case 12	1	2	1
Case 13	1	2	2

Histotype score definitions: 1 = epithelioid; 2 = biphasic; 3 = sarcomatoid. Atypie score means: 1 = milde; 2 = moderate; 3 = severe. Inflammatory infiltrate score definitions: 1 = positive; 2 = negative.

**Table 3:** Morphological characteristics of mesothelioma patients’ biopsies relative to proliferation and necrosis.

Patients	Necrosis	Mitotic Index-Pleura	Mitotic Index Adjusted	Nuclear Grade	Ki-67
Case 1	2	3	3	2	2
Case 2	1	3	3	3	2
Case 3	2	2	1	2	1
Case 4	1	3	3	3	2
Case 5	1	3	2	2	2
Case 6	1	3	3	3	1
Case 7	1	3	3	3	2
Case 8	2	2	1	1	1
Case 9	1	2	1	3	1
Case 10	2	3	2	3	2
Case 11	2	2	1	2	1
Case 12	2	2	1	2	1
Case 13	1	3	2	3	1

Necrosis score meanings: 1 = yes; 2 = no. Mitotic index-pleura score meanings: 1 = 1; 2 = 2–4; 3 = 5. Mitotic index adjusted meanings: 1 = 2–4; 2 = 5–9; 3 = >10. Nuclear grade score meanings: 1 = round uniform nuclei; 2 = irregular nuclear contours, small nucleoli; 3 = pleomorphism, markedly irregular nuclear contours and prominent nucleoli. Ki-67 score meanings: 1 = <20; 2 = >20.

**Table 4:** Histological characteristics of mesothelioma patients’ biopsies relative to BAP1, p16 and MTAP.

Patients	BAP1	IHC p16	FISH *p16*^a^	FISH *p16*^b^	FISH Del Homo	FISH Del Hetero	IHC MTAP
Case 1	2	1	2	1	2	2	2
Case 2	2	1	1	1	1	1	2
Case 3	2	2	3	0	2	1	1
Case 4	2	2	3	0	2	1	2
Case 5	2	2	3	0	2	1	1
Case 6	2	2	3	0	2	1	1
Case 7	1	2	3	0	2	1	2
Case 8	2	1	1	1	1	1	2
Case 9	2	1	2	1	2	2	1
Case 10	1	2	3	0	2	1	2
Case 11	2	1	2	1	2	2	2
Case 12	2	1	3	0	2	1	2
Case 13	2	1	2	1	2	2	2

BAP1 score meanings: 1 = positive; 2 = negative. IHC p16 score meanings: 1 = positive; 2 = negative. FISH *p16*^a^ score meanings: 1 = no del; 2 = del hetero; 3 = del homo. FISH *p16*^b^ score meanings: 0 = del omo; 1 = no del. FISH del homo score meanings: 1 = <20; 2 = >20. FISH del hetero meanings: 1 = <50; 2 = >50. IHC MTAP score meanings: 1 = negative; 2 = positive.

**Table 5:** TERT mRNA expression in mesothelioma patients’ biopsies.

Patients	Percentage of Cells in ‘1+’	Percentage of Cells in ‘2+’	Percentage of Cells in ‘3+’	Percentage of Cells in ‘4+’	Score
Case 1	40.55	3.47	0.00	0.00	2
Case 2	0.91	0.00	0.00	0.00	1
Case 3	53.90	24.76	1.66	0.17	4
Case 4	9.93	0.16	0.00	0.00	2
Case 5	4.80	0.00	0.00	0.00	1
Case 6	15.54	0.36	0.00	0.00	2
Case 7	6.34	0.04	0.00	0.00	2
Case 8	9.03	0.06	0.00	0.00	2
Case 9	6.52	0.00	0.00	0.00	1
Case 10	0.53	0.00	0.00	0.00	1
Case 11	6.31	0.00	0.00	0.00	1
Case 12	45.06	5.37	0.00	0.00	2
Case 13	7.75	0.00	0.00	0.00	1

The “Percentage of Cells in” indicates the percentage of cells that contained 1–3 dots per cell (1+), 4–9 dots per cell (2+), 10–15 dots per cell (3+) or ≥16 dots per cell (4+). The colors of the lines are unique for each case and correspond to the colors used for the graph in Figure 3.

**Table 6:** Pathological factors predictive of peritoneal mesothelioma patient survival. Univariate analysis using log-rank test.

Variables Months (95% CI)	No. of Cases Univariate	Mean Survival (Min–Max)	*p*-Value
*Histology*			
Epithelioid	7	30.86 (4–59)	0.0526
Biphasic	6	9.83 (1–46)	
Sarcomatoid	0	0	
*Mitotic count adjusted*			
2–4 = 1	5	33.20 (11–59)	≤0.0001
5–9 = 2	3	16.67 (1–45)	
>10 = 3	5	11.80 (1–47)	
*Ki67*			
<20	7	30.29 (1–59)	0.0666
>20	6	10.50 (1–47)	
*Sex*			
M	11	24.73 (1–59)	0.0152
F	2	1.50 (1–2)	
*BAP1*			
pos	2	1.50 (1–2)	0.0152
neg	11	24.73 (1–59)	
*IHC p16*			
pos	7	35.57 (2–59)	0.0022
neg	6	4.33 (1–11)	
*FISH p16*			
no del	2	15 (2–28)	0.0049
del homo	4	49.25 (45–59)	
del hetero	7	6.86 (1–22)	
*FISH p16*			
del homo	6	37.83 (2–59)	0.0044
no del	7	6.86 (1–22)	
*FISH p16 del hetero*			
<50	9	8.67 (1–28)	0.0019
>50	4	49.25 (45–59)	

## Data Availability

The original contributions presented in this study are included in the article. Further inquiries can be directed to the corresponding author.

## References

[1] Boussios S., Moschetta M., Karathanasi A., Tsiouris A.K., Kanellos F.S., Tatsi K., Katsanos K.H., Christodoulou D.K. (2018). Malignant peritoneal mesothelioma: Clinical aspects, and therapeutic perspectives. Ann. Gastroenterol..

[2] Greenbaum A., Alexander H.R. (2020). Peritoneal mesothelioma. Transl. Lung Cancer Res..

[3] Cerruto C.A., Brun E.A., Chang D., Sugarbaker P.H. (2006). Prognostic significance of histomorphologic parameters in diffuse malignant peritoneal mesothelioma. Arch. Pathol. Lab. Med..

[4] Bridda A., Padoan I., Mencarelli R., Frego M. (2007). Peritoneal mesothelioma: A review. Med. Gen. Med..

[5] Brenner J., Sordillo P.P., Magill G.B., Golbey R.B. (1981). Malignant peritoneal mesothelioma: Review of 25 patients. Am. J. Gastroenterol..

[6] Elias D., Bedard V., Bouzid T., Duvillard P., Kohneh-Sharhi N., Raynard B., Goere D. (2007). Malignant peritoneal mesothelioma: Treatment with maximal cytoreductive surgery plus intraperitoneal chemotherapy. Gastroenterol. Clin. Biol..

[7] Magge D., Zenati M.S., Austin F., Mavanur A., Sathaiah M., Ramalingam L., Jones H., Zureikat A.H., Holtzman M., Ahrendt S. (2014). Malignant peritoneal mesothelioma: Prognostic factors and oncologic outcome analysis. Ann. Surg. Oncol..

[8] Li C.Y., Kennedy T., Alexander H.R. (2022). Treatment of Patients with Malignant Peritoneal Mesothelioma. J. Clin. Med..

[9] Sugarbaker P.H. (2018). Update on the management of malignant peritoneal mesothelioma. Transl. Lung Cancer Res..

[10] Alaklabi S., Roy A.M., Skitzki J.J., Iyer R. (2023). Immunotherapy in malignant peritoneal mesothelioma (Review). Mol. Clin. Oncol..

[11] Kusamura S., Kepenekian V., Villeneuve L., Lurvink R.J., Govaerts K., De Hingh I., Moran B.J., Van der Speeten K., Deraco M., Glehen O. (2021). Peritoneal mesothelioma: PSOGI/EURACAN clinical practice guidelines for diagnosis, treatment and follow-up. Eur. J. Surg. Oncol..

[12] Brandl A., Westbrook S., Nunn S., Arbuthnot-Smith E., Mulsow J., Youssef H., Carr N., Tzivanakis A., Dayal S., Mohamed F. (2020). Clinical and surgical outcomes of patients with peritoneal mesothelioma discussed at a monthly national multidisciplinary team video-conference meeting. BJS Open.

[13] Benedetti S., Nuvoli B., Catalani S., Galati R. (2015). Reactive oxygen species a double-edged sword for mesothelioma. Oncotarget.

[14] Karpes J.B., Shamavonian R., Dewhurst S., Cheng E., Wijayawardana R., Ahmadi N., Morris D.L. (2023). Malignant Peritoneal Mesothelioma: An In-Depth and Up-to-Date Review of Pathogenesis, Diagnosis, Management and Future Directions. Cancers.

[15] Hung Y.P., Dong F., Torre M., Crum C.P., Bueno R., Chirieac L.R. (2020). Molecular characterization of diffuse malignant peritoneal mesothelioma. Mod. Pathol..

[16] Hiltbrunner S., Fleischmann Z., Sokol E.S., Zoche M., Felley-Bosco E., Curioni-Fontecedro A. (2022). Genomic landscape of pleural and peritoneal mesothelioma tumours. Br. J. Cancer.

[17] Fortarezza F., Pezzuto F., Marzullo A., Cavone D., Romano D.E., d’Amati A., Serio G., Vimercati L. (2022). Molecular Pathways in Peritoneal Mesothelioma: A Minireview of New Insights. Front. Oncol..

[18] Singhi A.D., Krasinskas A.M., Choudry H.A., Bartlett D.L., Pingpank J.F., Zeh H.J., Luvison A., Fuhrer K., Bahary N., Seethala R.R. (2016). The prognostic significance of BAP1, NF2, and CDKN2A in malignant peritoneal mesothelioma. Mod. Pathol..

[19] Dietz M.V., van Kooten J.P., Paats M.S., Aerts J., Verhoef C., Madsen E.V.E., Dubbink H.J., von der Thusen J.H. (2023). Molecular alterations and potential actionable mutations in peritoneal mesothelioma: A scoping review of high-throughput sequencing studies. ESMO Open.

[20] Prasad K.N., Wu M., Bondy S.C. (2017). Telomere shortening during aging: Attenuation by antioxidants and anti-inflammatory agents. Mech. Ageing Dev..

[21] Dratwa M., Wysoczanska B., Lacina P., Kubik T., Bogunia-Kubik K. (2020). TERT-Regulation and Roles in Cancer Formation. Front. Immunol..

[22] Hanahan D. (2022). Hallmarks of Cancer: New Dimensions. Cancer Discov..

[23] Gaspar T.B., Sa A., Lopes J.M., Sobrinho-Simoes M., Soares P., Vinagre J. (2018). Telomere Maintenance Mechanisms in Cancer. Genes.

[24] Udutha S., Taglang C., Batsios G., Gillespie A.M., Tran M., Ronen S.M., Ten Hoeve J., Graeber T.G., Viswanath P. (2023). Telomerase reverse transcriptase induces targetable alterations in glutathione and nucleotide biosynthesis in glioblastomas. bioRxiv.

[25] Ahmad F., Dixit D., Sharma V., Kumar A., Joshi S.D., Sarkar C., Sen E. (2016). Nrf2-driven TERT regulates pentose phosphate pathway in glioblastoma. Cell Death Dis..

[26] Viswanath P., Batsios G., Ayyappan V., Taglang C., Gillespie A.M., Larson P.E.Z., Luchman H.A., Costello J.F., Pieper R.O., Ronen S.M. (2021). Metabolic imaging detects elevated glucose flux through the pentose phosphate pathway associated with TERT expression in low-grade gliomas. Neuro-Oncology.

[27] Batsios G., Taglang C., Tran M., Stevers N., Barger C., Gillespie A.M., Ronen S.M., Costello J.F., Viswanath P. (2022). Deuterium Metabolic Imaging Reports on TERT Expression and Early Response to Therapy in Cancer. Clin. Cancer Res..

[28] Ahmad F., Patrick S., Sheikh T., Sharma V., Pathak P., Malgulwar P.B., Kumar A., Joshi S.D., Sarkar C., Sen E. (2017). Telomerase reverse transcriptase (TERT)—Enhancer of zeste homolog 2 (EZH2) network regulates lipid metabolism and DNA damage responses in glioblastoma. J. Neurochem..

[29] Cangelosi D., Morini M., Zanardi N., Sementa A.R., Muselli M., Conte M., Garaventa A., Pfeffer U., Bosco M.C., Varesio L. (2020). Hypoxia Predicts Poor Prognosis in Neuroblastoma Patients and Associates with Biological Mechanisms Involved in Telomerase Activation and Tumor Microenvironment Reprogramming. Cancers.

[30] Petrova V., Annicchiarico-Petruzzelli M., Melino G., Amelio I. (2018). The hypoxic tumour microenvironment. Oncogenesis.

[31] Ramachandran D., Mao Q., Liao D., Kamal M., Schurmann P., Eisenblatter R., Geffers R., Balint B., Lecompte L., Servant N. (2025). Methylation, Gene Expression, and Risk Genotypes at the TERT-CLPTM1L Locus in Cervical Cancer. Mol. Carcinog..

[32] Fleisig H.B., Hukezalie K.R., Thompson C.A.H., Au-Yeung T.T.T., Ludlow A.T., Zhao C.R., Wong J.M.Y. (2016). Telomerase reverse transcriptase expression protects transformed human cells against DNA-damaging agents, and increases tolerance to chromosomal instability. Oncogene.

[33] Yuan X., Xu D. (2019). Telomerase Reverse Transcriptase (TERT) in Action: Cross-Talking with Epigenetics. Int. J. Mol. Sci..

[34] Im E., Yoon J.B., Lee H.W., Chung K.C. (2017). Human Telomerase Reverse Transcriptase (hTERT) Positively Regulates 26S Proteasome Activity. J. Cell Physiol..

[35] Chen J., Nelson C., Wong M., Tee A.E., Liu P.Y., La T., Fletcher J.I., Kamili A., Mayoh C., Bartenhagen C. (2021). Targeted Therapy of TERT-Rearranged Neuroblastoma with BET Bromodomain Inhibitor and Proteasome Inhibitor Combination Therapy. Clin. Cancer Res..

[36] Zou Y., Cong Y.S., Zhou J. (2020). Implications of telomerase reverse transcriptase in tumor metastasis. BMB Rep..

[37] Xie L., Yin W., Tang F., He M. (2023). Pan-Cancer analysis of TERT and Validation in Osteosarcoma Cell Lines. Biochem. Biophys. Res. Commun..

[38] Gao M., Lin Y., Liu X., Zhao Z., Zhu Z., Zhang H., Ban Y., Bie Y., He X., Sun X. (2021). TERT Mutation Is Accompanied by Neutrophil Infiltration and Contributes to Poor Survival in Isocitrate Dehydrogenase Wild-Type Glioma. Front. Cell Dev. Biol..

[39] Giunco S., Rampazzo E., Celeghin A., Petrara M.R., De Rossi A. (2015). Telomere and Telomerase in Carcinogenesis: Their Role as Prognostic Biomarkers. Curr. Pathobiol. Rep..

[40] Fu G., Chazen R.S., Monteiro E., Vescan A., Freeman J.L., Witterick I.J., MacMillan C. (2023). Facilitation of Definitive Cancer Diagnosis With Quantitative Molecular Assays of BRAF V600E and TERT Promoter Variants in Patients With Thyroid Nodules. JAMA Netw. Open.

[41] Boutko A., Hagstrom M., Lampley N., Roth A., Olivares S., Dhillon S., Fumero-Velazquez M., Benton S., Zhao J., Zhang B. (2023). PRAME Immunohistochemical Expression and TERT Promoter Mutational Analysis as Ancillary Diagnostic Tools for Differentiating Proliferative Nodules From Melanoma Arising in Congenital Nevi. Am. J. Dermatopathol..

[42] Michalkova R., Safanda A., Hajkova N., Hojny J., Krkavcova E., Kendall Bartu M., Svajdler M., Shatokhina T., Laco J., Matej R. (2024). The Molecular Landscape of 227 Adult Granulosa Cell Tumors of the Ovary: Insights into the Progression from Primary to Recurrence. Lab. Investig..

[43] Bertorelle R., Briarava M., Rampazzo E., Biasini L., Agostini M., Maretto I., Lonardi S., Friso M.L., Mescoli C., Zagonel V. (2013). Telomerase is an independent prognostic marker of overall survival in patients with colorectal cancer. Br. J. Cancer.

[44] Rampazzo E., Del Bianco P., Bertorelle R., Boso C., Perin A., Spiro G., Bergamo F., Belluco C., Buonadonna A., Palazzari E. (2018). The predictive and prognostic potential of plasma telomerase reverse transcriptase (TERT) RNA in rectal cancer patients. Br. J. Cancer.

[45] Blanco-Garcia L., Ruano Y., Blanco Martinez-Illescas R., Cubo R., Jimenez Sanchez P., Sanchez-Arevalo Lobo V.J., Riveiro Falkenbach E., Ortiz Romero P., Garrido M.C., Rodriguez Peralto J.L. (2023). pTERT C250T mutation: A potential biomarker of poor prognosis in metastatic melanoma. Heliyon.

[46] Sauter J.L., Dacic S., Galateau-Salle F., Attanoos R.L., Butnor K.J., Churg A., Husain A.N., Kadota K., Khoor A., Nicholson A.G. (2022). The 2021 WHO Classification of Tumors of the Pleura: Advances Since the 2015 Classification. J. Thorac. Oncol..

[47] Scattone A., Serio G., Marzullo A., Nazzaro P., Corsi F., Cocca M.P., Mattoni M., Punzi A., Gentile M., Buonadonna A.L. (2012). High Wilms’ tumour gene (WT1) expression and low mitotic count are independent predictors of survival in diffuse peritoneal mesothelioma. Histopathology.

[48] Travis W.D., Brambilla E., Nicholson A.G., Yatabe Y., Austin J.H.M., Beasley M.B., Chirieac L.R., Dacic S., Duhig E., Flieder D.B. (2015). The 2015 World Health Organization Classification of Lung Tumors: Impact of Genetic, Clinical and Radiologic Advances Since the 2004 Classification. J. Thorac. Oncol..

[49] Dacic S., Kothmaier H., Land S., Shuai Y., Halbwedl I., Morbini P., Murer B., Comin C., Galateau-Salle F., Demirag F. (2008). Prognostic significance of p16/cdkn2a loss in pleural malignant mesotheliomas. Virchows. Arch..

[50] Chapel D.B., Schulte J.J., Berg K., Churg A., Dacic S., Fitzpatrick C., Galateau-Salle F., Hiroshima K., Krausz T., Le Stang N. (2020). MTAP immunohistochemistry is an accurate and reproducible surrogate for CDKN2A fluorescence in situ hybridization in diagnosis of malignant pleural mesothelioma. Mod. Pathol..

[51] Hida T., Hamasaki M., Matsumoto S., Sato A., Tsujimura T., Kawahara K., Iwasaki A., Okamoto T., Oda Y., Honda H. (2017). Immunohistochemical detection of MTAP and BAP1 protein loss for mesothelioma diagnosis: Comparison with 9p21 FISH and BAP1 immunohistochemistry. Lung Cancer.

[52] Pezzuto F., Serio G., Fortarezza F., Scattone A., Caporusso C., Punzi A., Cavone D., Pennella A., Marzullo A., Vimercati L. (2020). Prognostic Value of Ki67 Percentage, WT-1 Expression and p16/CDKN2A Deletion in Diffuse Malignant Peritoneal Mesothelioma: A Single-Centre Cohort Study. Diagnostics.

[53] Cigognetti M., Lonardi S., Fisogni S., Balzarini P., Pellegrini V., Tironi A., Bercich L., Bugatti M., Rossi G., Murer B. (2015). BAP1 (BRCA1-associated protein 1) is a highly specific marker for differentiating mesothelioma from reactive mesothelial proliferations. Mod. Pathol..

[54] Marczyk V.R., Maia A.L., Goemann I.M. (2024). Distinct transcriptional and prognostic impacts of TERT promoter mutations C228T and C250T in papillary thyroid carcinoma. Endocr. Relat. Cancer.

[55] Melo M., da Rocha A.G., Vinagre J., Batista R., Peixoto J., Tavares C., Celestino R., Almeida A., Salgado C., Eloy C. (2014). TERT promoter mutations are a major indicator of poor outcome in differentiated thyroid carcinomas. J. Clin. Endocrinol. Metab..

[56] Dome J.S., Bockhold C.A., Li S.M., Baker S.D., Green D.M., Perlman E.J., Hill D.A., Breslow N.E. (2005). High telomerase RNA expression level is an adverse prognostic factor for favorable-histology Wilms’ tumor. J. Clin. Oncol..

[57] Chae M., Lee J.H., Park J.H., Keum D.Y., Jung H., Lee Y., Lee D.H. (2024). Different Role of TRF1 and TRF2 Expression in Non-Small Cell Lung Cancers. OncoTargets Ther..

[58] Koneru B., Lopez G., Farooqi A., Conkrite K.L., Nguyen T.H., Macha S.J., Modi A., Rokita J.L., Urias E., Hindle A. (2020). Telomere Maintenance Mechanisms Define Clinical Outcome in High-Risk Neuroblastoma. Cancer Res..

[59] Atout S., Shurrab S., Loveridge C. (2022). Evaluation of the Suitability of RNAscope as a Technique to Measure Gene Expression in Clinical Diagnostics: A Systematic Review. Mol. Diagn. Ther..

[60] Annese T., Tamma R., Ribatti D. (2022). RNAscope for VEGF-A Detection in Human Tumor Bioptic Specimens. Methods. Mol. Biol..

[61] Annese T., Tamma R., De Giorgis M., Ruggieri S., Maiorano E., Specchia G., Ribatti D. (2020). RNAscope dual ISH-IHC technology to study angiogenesis in diffuse large B-cell lymphomas. Histochem. Cell Biol..

[62] Tamma R., Annese T., Ruggieri S., Marzullo A., Nico B., Ribatti D. (2018). VEGFA and VEGFR2 RNAscope determination in gastric cancer. J. Mol. Histol..

[63] Momeni-Boroujeni A., Yousefi E., Gupta S., Benayed R., Berger M.F., Ladanyi M., Monroe R., Kim J., Jungbluth A., Weigelt B. (2022). Evaluation of TERT mRNA expression using RNAscope(R): A potential histopathologic diagnostic and prognostic tool. Pathol. Res. Pract..

[64] Ghosh A., Saginc G., Leow S.C., Khattar E., Shin E.M., Yan T.D., Wong M., Zhang Z., Li G., Sung W.K. (2012). Telomerase directly regulates NF-kappaB-dependent transcription. Nat. Cell Biol..

[65] Xue H., Hu Z., Liu S., Zhang S., Yang W., Li J., Yan C., Zhang J., Zhang J., Lei X. (2024). The mechanism of NF-kappaB-TERT feedback regulation of granulosa cell apoptosis in PCOS rats. PLoS ONE.

[66] Pineiro-Hermida S., Bosso G., Sanchez-Vazquez R., Martinez P., Blasco M.A. (2023). Telomerase deficiency and dysfunctional telomeres in the lung tumor microenvironment impair tumor progression in NSCLC mouse models and patient-derived xenografts. Cell Death Differ..

[67] Zanetti M. (2017). A second chance for telomerase reverse transcriptase in anticancer immunotherapy. Nat. Rev. Clin. Oncol..

[68] Lopes-Bastos B., Nabais J., Ferreira T., Allavena G., El Mai M., Bird M., Targen S., Tattini L., Kang D., Yue J.X. (2024). The absence of telomerase leads to immune response and tumor regression in zebrafish melanoma. Cell Rep..

[69] Kovalenko O.A., Caron M.J., Ulema P., Medrano C., Thomas A.P., Kimura M., Bonini M.G., Herbig U., Santos J.H. (2010). A mutant telomerase defective in nuclear-cytoplasmic shuttling fails to immortalize cells and is associated with mitochondrial dysfunction. Aging Cell.

[70] Xian S., Dosset M., Castro A., Carter H., Zanetti M. (2023). Transcriptional analysis links B cells and TERT expression to favorable prognosis in head and neck cancer. PNAS Nexus.

[71] Chapel D.B., Schulte J.J., Absenger G., Attanoos R., Brcic L., Butnor K.J., Chirieac L., Churg A., Galateau-Salle F., Hiroshima K. (2021). Malignant peritoneal mesothelioma: Prognostic significance of clinical and pathologic parameters and validation of a nuclear-grading system in a multi-institutional series of 225 cases. Mod. Pathol..

[72] Pezzuto F., Vimercati L., Fortarezza F., Marzullo A., Pennella A., Cavone D., Punzi A., Caporusso C., d’Amati A., Lettini T. (2021). Evaluation of prognostic histological parameters proposed for pleural mesothelioma in diffuse malignant peritoneal mesothelioma. A short report. Diagn. Pathol..

[73] Alpert N., van Gerwen M., Taioli E. (2020). Epidemiology of mesothelioma in the 21(st) century in Europe and the United States, 40 years after restricted/banned asbestos use. Transl. Lung Cancer Res..

[74] Alpert N., van Gerwen M., Flores R., Taioli E. (2020). Gender Differences in Outcomes of Patients With Mesothelioma. Am. J. Clin. Oncol..

[75] Musk A.W., Olsen N., Alfonso H., Reid A., Mina R., Franklin P., Sleith J., Hammond N., Threlfall T., Shilkin K.B. (2011). Predicting survival in malignant mesothelioma. Eur. Respir. J..

[76] Leblay N., Lepretre F., Le Stang N., Gautier-Stein A., Villeneuve L., Isaac S., Maillet D., Galateau-Salle F., Villenet C., Sebda S. (2017). BAP1 Is Altered by Copy Number Loss, Mutation, and/or Loss of Protein Expression in More Than 70% of Malignant Peritoneal Mesotheliomas. J. Thorac. Oncol..

[77] Baumann F., Flores E., Napolitano A., Kanodia S., Taioli E., Pass H., Yang H., Carbone M. (2015). Mesothelioma patients with germline BAP1 mutations have 7-fold improved long-term survival. Carcinogenesis.

[78] Hwang H.C., Sheffield B.S., Rodriguez S., Thompson K., Tse C.H., Gown A.M., Churg A. (2016). Utility of BAP1 Immunohistochemistry and p16 (CDKN2A) FISH in the Diagnosis of Malignant Mesothelioma in Effusion Cytology Specimens. Am. J. Surg. Pathol..

[79] Rossi G., Righi L., Barbisan F., Tiseo M., Spagnolo P., Grosso F., Pisapia P., Malapelle U., Sculco M., Dianzani I. (2024). BAP1 Loss, Nuclear Grading, and Nonepithelioid Features in the Diagnosis of Mesothelioma in Italy: Nevermore without the Pathology Report. J. Pers. Med..

[80] Kotiyal S., Evason K.J. (2021). Exploring the Interplay of Telomerase Reverse Transcriptase and beta-Catenin in Hepatocellular Carcinoma. Cancers.

[81] Ningarhari M., Caruso S., Hirsch T.Z., Bayard Q., Franconi A., Vedie A.L., Noblet B., Blanc J.F., Amaddeo G., Ganne N. (2021). Telomere length is key to hepatocellular carcinoma diversity and telomerase addiction is an actionable therapeutic target. J. Hepatol..

[82] Borczuk A.C., Taub R.N., Hesdorffer M., Hibshoosh H., Chabot J.A., Keohan M.L., Alsberry R., Alexis D., Powell C.A. (2005). P16 loss and mitotic activity predict poor survival in patients with peritoneal malignant mesothelioma. Clin. Cancer Res..

[83] Shim H.S., Iaconelli J., Shang X., Li J., Lan Z.D., Jiang S., Nutsch K., Beyer B.A., Lairson L.L., Boutin A.T. (2024). TERT activation targets DNA methylation and multiple aging hallmarks. Cell.

